# Populations of Radial Glial Cells Respond Differently to Reelin and Neuregulin1 in a Ferret Model of Cortical Dysplasia

**DOI:** 10.1371/journal.pone.0013709

**Published:** 2010-10-28

**Authors:** Sylvie Poluch, Sharon L. Juliano

**Affiliations:** 1 Anatomy, Physiology, and Genetics, Uniformed Services University, Bethesda, Maryland, United States of America; 2 Neuroscience, Uniformed Services University, Bethesda, Maryland, United States of America; Ecole Normale Supérieure de Lyon, France

## Abstract

Radial glial cells play an essential role during corticogenesis through their function as neural precursors and guides of neuronal migration. Both reelin and neuregulin1 (NRG1) maintain the radial glial scaffold; they also induce expression of Brain Lipid Binding Protein (BLBP), a well known marker of radial glia. Although radial glia in normal ferrets express both vimentin and BLBP, this coexpression diverges at P3; vimentin is expressed in the radial glial processes, while BLBP appears in cells detached from the ventricular zone. Our lab developed a model of cortical dysplasia in the ferret, resulting in impaired migration of neurons into the cortical plate and disordered radial glia. This occurs after exposure to the antimitotic methylazoxymethanol (MAM) on the 24th day of development (E24). Ferrets treated with MAM on E24 result in an overall decrease of BLBP expression; radial glia that continue to express BLBP, however, show only mild disruption compared with the strongly disrupted vimentin expressing radial glia. When E24 MAM-treated organotypic slices are exposed to reelin or NRG1, the severely disrupted vimentin+ radial glial processes are repaired but the slightly disordered BLBP+ processes are not. The realignment of vimentin+ processes was linked with an increase of their BLBP expression. BLBP expressing radial glia are distinguished by being both less affected by MAM treatment and by attempts at repair. We further investigated the effects induced by reelin and found that signaling was mediated via VLDLR/Dab1/Pi3K activation while NRG1 signaling was mediated via erbB3/erbB4/Pi3K. We then tested whether radial glial repair correlated with improved neuronal migration. Repairing the radial glial scaffold is not sufficient to restore neuronal migration; although reelin improves migration of neurons toward the cortical plate signaling through ApoER2/Dab1/PI3K activation, NRG1 does not.

## Introduction

The developing cerebral cortex contains a transient elongated population, the radial glial cells, which play an essential role through their function as guides of neuronal migration and neural precursors [1], [2]. Radial glial cells appear to be particularly vulnerable to prenatal environmental insults: alcohol [3], methyl mercury [4], ionizing radiation [5] or cytotoxins such as methylazoxymethanol (MAM) [6]; as a result, radial glia prematurely differentiate into astrocytes. Disruption of the radial glial scaffold causes neural migration disorders, often leading to cortical dysplasia, which underlies many syndromes including mental retardation, epilepsy, schizophrenia, and autism [7], [8], [9].

In rodents, neurogenesis and neuronal migration are largely complete at birth, at which time radial glial cells transform into astrocytes [10], [11]. Ferrets, on the other hand, have a protracted development and the radial glial scaffold is maintained until 3 weeks after birth; neurogenesis of upper layer neurons (layers 2 and 3) occurs postnatally [11], [12], [13], [14], [15]. Ferrets are also the smallest mammals with a convoluted cortex [16]. Proliferation of intermediate progenitor cells in ferrets occurs for an extended period compared to rats and may underlie the tangential expansion of the gyrencephalic cerebral cortex in carnivores and primates [17], [18]. Recently, Fietz et al. [19] proposed that outer subventricular progenitors have a fundamental role in cortical expansion of gyrencephalic brains in ferrets as well as in humans. These distinctions emphasize that it is important to involve more complex mammals like ferrets in developmental studies as fundamental processes can differ between species (e.g. [15]).

We developed a model of cortical dysplasia in the ferret, in which radial glia display severe disruption and undergo premature differentiation into astrocytes [20], [21]. Exposing embryos *in utero* to the antimitotic MAM on the 24^th^ day of development (E24) disrupts early cortical development, resulting in a thin and poorly laminated cortex, where neurons migrating radially and tangentially fail to reach the cortical plate [20], [21], [22].

Radial glia are a diverse population and express a number of specific markers at different times and throughout development. They also respond to different signals, which regulate their status as both neural progenitor cells and a scaffold for migration [2]. Neuregulin1 (NRG1) is crucial to maintaining a normal radial glial scaffold [23], [24] and signals *via* members of the ErbB family of receptor tyrosine kinases [25], [26], [27]. Radial glial disruption in E24 MAM treated cortex is likely to be caused in part by reduction of NRG1, because exogenous replacement results in realignment in E24 MAM treated organotypic slices [28].

Reelin is another key protein active during cortical development as the lack of reelin results in aberrant migration of cortical neurons and misaligned radial glial cells [29], [30], [31], [32]. Exogenous reelin promotes radial glial extension and rescues the radial glial scaffold in reeler hippocampus [33]. Reelin signaling requires binding to receptors of the lipoprotein family, very low density-lipoprotein (VLDLR) and the apolipoprotein E receptor (ApoER2), which triggers tyrosine phosphorylation of the cytoplasmic adapter protein Disabled-1 (Dab1) [34], [35], [36]. Dab1 is expressed in cortical neurons [37] as well as in radial glial cells [38]. In E24 MAM treated ferrets an exogenous source of reelin secreted at the pial surface improves neuronal migration as well as radial glial morphology [39].

In addition to radial process extension, reelin and NRG1 promote expression of Brain Lipid Binding Protein (BLBP) in cortical radial glia [40], [41], [32]. Although the function of BLBP during cortical development is not fully understood, BLBP expression strongly correlates with the migration of neurons along the radial glia [21]. BLBP appears to be required for radial process elongation, since the addition of anti-BLBP antibodies inhibit this process [42].

We show here that in normal newborn ferrets, vimentin and BLBP are strongly expressed in radial glia. In our ferret model of cortical dysplasia, the expression of BLBP in radial glia is decreased after MAM treatment; however the remaining BLBP+ radial glial cells are relatively spared from disruption compared with the severely disorganized vimentin+ cells. Both reelin and NRG1 realign the disorganized vimentin+ radial glial cells. Although the morphology of BLBP+ cells was not improved from their mild disruption after these treatments, the expression of BLBP was increased. This suggests that at least two distinct populations of radial glial cells exist in ferrets that respond differently to damage and attempts at repair. Exogenous reelin improves not only the radial glial scaffold but also radial migration toward the cortical plate whereas NRG1 has no effect on neuronal migration. In addition, distinct signaling elements appear to initiate movement out of the ventricular zone, but do not play a role in allowing further movement toward the cortical plate.

## Materials and Methods

### Ethics Statement

The use of animals and the methods of this study were approved by the Institutional Animal Care and Use Committee (IACUC) at USUHS and under Animal Welfare Assurance number A3448-01. The experiments were performed at an AAALAC accredited institute.

### Animals

Timed pregnant ferrets (*Mustella putorius*) were purchased from Marshall Farms (New Rose, NY); ferret kits are born after 41 days of gestation. Pregnant ferrets, anaesthetized with isofluorane using a mask (5%), were injected intraperitoneally (IP) with methylazoxy methanol acetate (MAM, Midwest Research Institute, Kansas City, MO, 14 mg/kg) diluted in a sterile saline buffer. Normal and MAM treated fetuses at E27, E33, or E38-E40 were obtained by caesarean section under sterile conditions using isofluorane anesthesia under the supervision of a veterinarian. We also used normal and MAM treated newborn kits (postnatal day 0, P0), as well as normal P3, P14, and P28 normal ferrets, which were anesthetized with an IP injection of pentobarbital sodium (50 mg/kg) prior to brain removal.

### Organotypic culture

Brains obtained from E39-E40 embryos were cut under sterile conditions into 400 µm thick coronal slices using a tissue chopper (Stoelting, Wood Dale, IL). During the dissection, brains and slices were perfused with cold, oxygenated artificial cerebrospinal fluid (containing in mM: CaCl2 2.4, KCl 3.2, MgSO4 1.2, NaCl 124, NaHCO3 26, NaH2PO4 1.2, glucose 10). Coronal cortical slices containing the somatosensory cortex [13], [20] were placed on inserts (Millipore, Bedford, MA) in 6-well plates using MEM medium (Gibco, Carlsbad, CA) containing 10% decomplemented horse serum (Gibco) and 4% G1,2 solution (0.5 mg/mL gentamycin, 15% glucose, 50 mM L-glutamine). A number of organotypic slices were incubated for 1 hour in medium supplemented with BrdU (100 µg/ml), which was removed and then replaced with fresh medium. After 2 days in culture (DIC) in an incubator (95% CO_2_; 37°C), the organotypic slices were fixed for 2 hours by immersion in 4% phosphate buffered paraformaldehyde. In some cases, fixed slices were also cryoprotected and subsequently re-sectioned at 14 µm using a cryostat.

### Coculture of organotypic ferret slices with HEK cells

A number of MAM treated slices were co-cultured with: (1) HEK 293T cells, transfected with the mouse reelin cDNA construct pCrl [43], which produces and secretes the full length reelin protein [44], [39], (2) HEK 293T cells, transfected with the type I NRG1 encoding plasmid (NRG1-Ig), which secretes the full length of the isoform type I NRG1 [45] and (3) HEK 293T cells, transfected with the type III NRG1 (NRG1-CRD), which express a non-secreted/membrane type III NRG1 [45]. Prior to the coculture, HEK 293T cells were cultured in Dulbecco's modified Eagle's medium (MEDIATECH Inc., Herndon, VA) (control HEK cells) supplemented with Geneticin (G418, 0.5 mg/ml) (reelin+, NRG1-Ig, or NRG1-CRD HEK cells). The HEK cells were placed in Matrigel (BD Biosciences, Bedford, MA) and positioned next to the pial surface [39].

### Drugs and chronic treatments

In some experiments, the medium was supplemented with recombinant mouse reelin (1 nM, US Biological, Swampscott, MA) corresponding to the central fragment. We also used recombinant NRG1 (1 nM, R&D systems, Minneapolis, MN) obtained from the Human DNA sequence encoding the EGF domain of NRG1 β1. To further understand the effects mediated by reelin or NRG1, the culture medium was complemented with pathway inhibitors such as: LY294002 (inhibitor of PI3K; 50 µM, Calbiochem, San Diego, CA), PP2 (a Src kinases inhibitor; 10 µM, Calbiochem), TDZD-8 (a GSK-3β Inhibitor I, 56 µM, Calbiochem), or SP600125 (a JNK Inhibitor II, 10 µM, Calbiochem). To block ApoER2 and VLDLR, we used human recombinant RAP (300 nM, Calbiochem). NRG1 signaling was inhibited by using blocking antibodies for NRG1 receptors, erbB-3 or erbB-4 (20 µg/ml, LabVision, Fremont, CA) (See Figure S1).

### Immunohistochemistry

For fluorescence immunocytochemistry, slices were incubated overnight at 4°C with mouse IgG monoclonal antibodies against: vimentin clone V9 (1/100, Sigma), MAP2abc (1/200, Sigma) or rabbit polyclonal antibodies against: BLBP (1/300, Chemicon and Abcam) and GABA (1/300, Sigma). After washes in PBS, the corresponding secondary antibodies were used (anti-rabbit, or anti-mouse Alexa-488 or Alexa-546, 1/200, Molecular Probes). The sections were washed in PBS and mounted in Mowiol.

### BrdU immunoreactivity

The fixed slices were placed in 70% cold ethanol for 10 minutes at 4°C, followed by 1 hour in 2N HCl at 37°C. Slices were then placed in borate buffer (pH 8.5) and washed in PBS. The following antibodies were used: anti-rat BrdU (1/100, Becton Dickinson, Franklin Lakes, NJ) and goat anti-rat IgG conjugated with CY2 (1/200, Jackson ImmunoResearch West Grove, PA) or goat anti-rat IgG Alexa-488 (1/200, Molecular Probes).

### Quantification of BrdU immunoreactive cells

To determine the ability of cells to migrate in organotypic cultures of either normal, E24 MAM treated cortex alone, or after coculture with HEK 293T cells embedded in Matrigel (as describe above), we plotted the distribution of BrdU+ cells after 2 DIC. Boundaries were drawn indicating the pia of a ferret slice and the outer edge of the VZ. This region was divided into 3 equal bins for each coculture and the number of cells per bin counted in a slab 500 µm in width in the somatosensory cortex. The bins correspond to the intermediate zone close to the VZ (*i.e.*, the lowest part of the intermediate zone, IZ_L_), a region in the IZ, but closer to the cortical plate (*i.e.*, the upper part of the intermediate zone, IZ_U_), and the region corresponding to the cortical plate (CP) (Figure 5c–d and Figure 7c–d). Histograms were made to indicate the position of BrdU+ cells across animals in each condition. To compare across samples, the number of cells/bin were calculated as a percent of the total number of cells in each slice.

### Quantification of radial glial morphology and phenotype

MAM treatment leads to early radial glial differentiation, which can be reversed by treatment with exogenous NRG1 or reelin [28], [39]. To quantify the change in morphology, treated and untreated E24 MAM slices were double labeled for 2 specific radial glial markers, BLBP and vimentin. All data were collected from the somatosensory cortex. The angle of deviation for each marker was measured as described previously [21], [28] using Image Tool (UTHSCSA, San Antonio, Texas). To determine the phenotype of radial glial cells in normal or MAM treated cortex, the number of processes expressing vimentin as well as BLBP, or only vimentin, or only BLBP was computed. Since radial glial cells can have several vertical processes, the data refer to radial glial processes and not radial glial cells. The result is expressed as a percentage of processes vimentin+ BLBP+ or BLBP- vimentin+ or vimentin- BLBP+. We used a 25X objective on a microscope equipped with an Apotome to acquire multiple *z*-stack images (at least 5 *z*-sections, with ∼5 µm interval), which were collapsed into a single image; the degrees of deviation and the phenotype of radial glial processes were measured in a 250 µm^2^ zone within the cortical plate. On average, this zone contains 57 radial glial processes.

### Statistical Analysis

A total of 115 MAM treated embryos were obtained from 16 pregnant ferrets and 27 normal ferret embryos or kits obtained from 13 pregnant ferrets. All data are obtained from at least two independent experiments from different litters. For all data, a 2 way ANOVA was conducted followed by a Holm-Sidak test for comparisons between groups. Statistical analyses were performed using SigmaStat (Systat Software, Inc, Chicago, Illinois).

### Image Acquisition

For the acquisition of fluorescence images, we used an Axiovert 200 microscope (Zeiss) equipped with an Apotome and Axiovision 4.7.

## Results

### Radial Glial phenotype in embryonic and postnatal development in normal ferret

**Figure 1:**
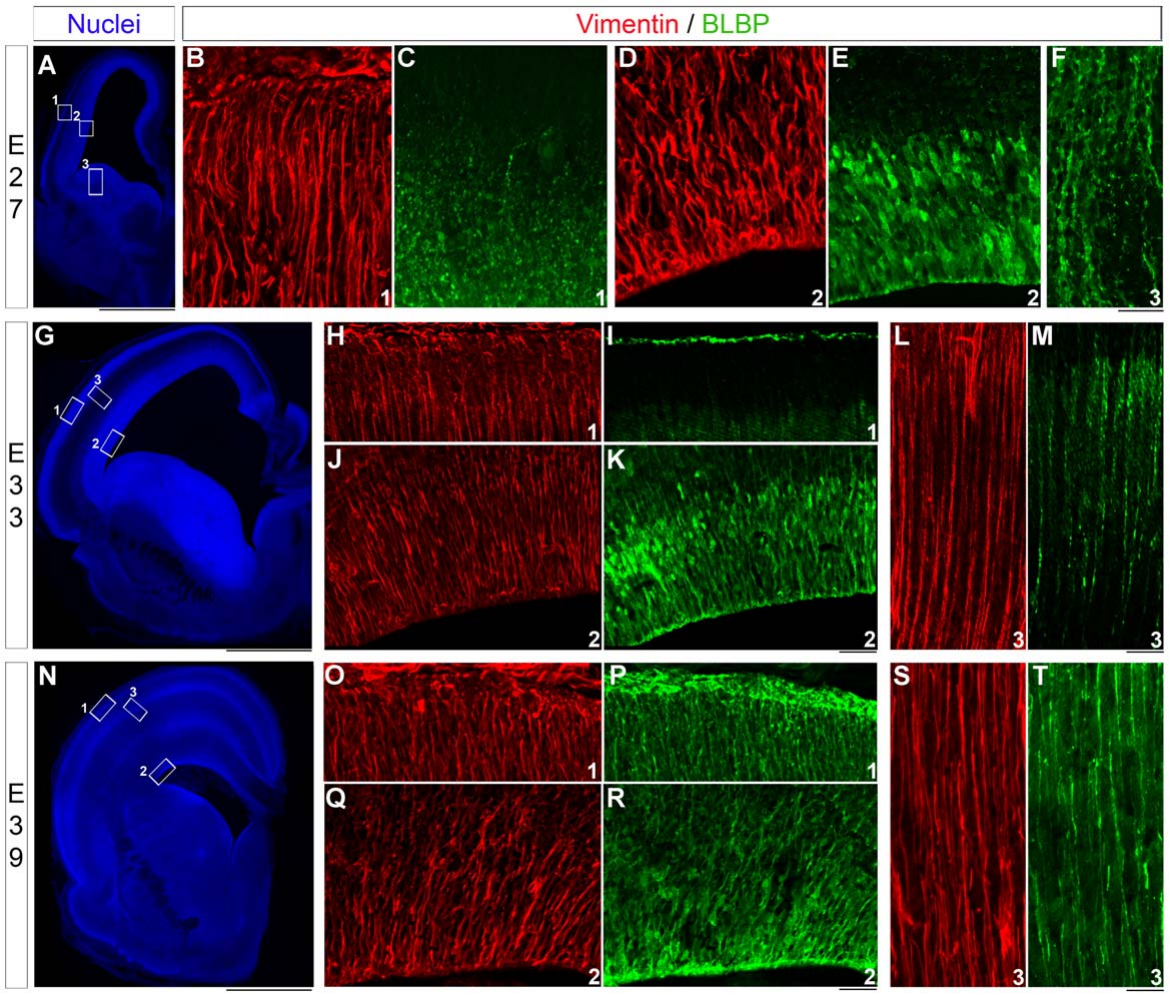
Expression of vimentin and BLBP during embryonic cortical development in normal ferrets. Immunostaining against vimentin (red) and BLBP (green) on coronal sections at E27 (**A–F**), E33 (**G–M**), and E39 (**N–T**). Nuclear staining with bisbenzimide (**A**,**G**,**N** in blue). At E27 vimentin immunoreactivity occurs in the cortical ventricular zone as well as in radial glial processes (**B**,**D**); BLBP, however is only found in the cortical ventricular zone (**E**) and in radial glial processes in the ganglionic eminence (**F**). E: embryonic day. Scale Bar: 50 µm (B–E,H–K,O–R), 100 µm (A,F,L,M,S,T) and 1000 µm (G,N).

**Figure 2:**
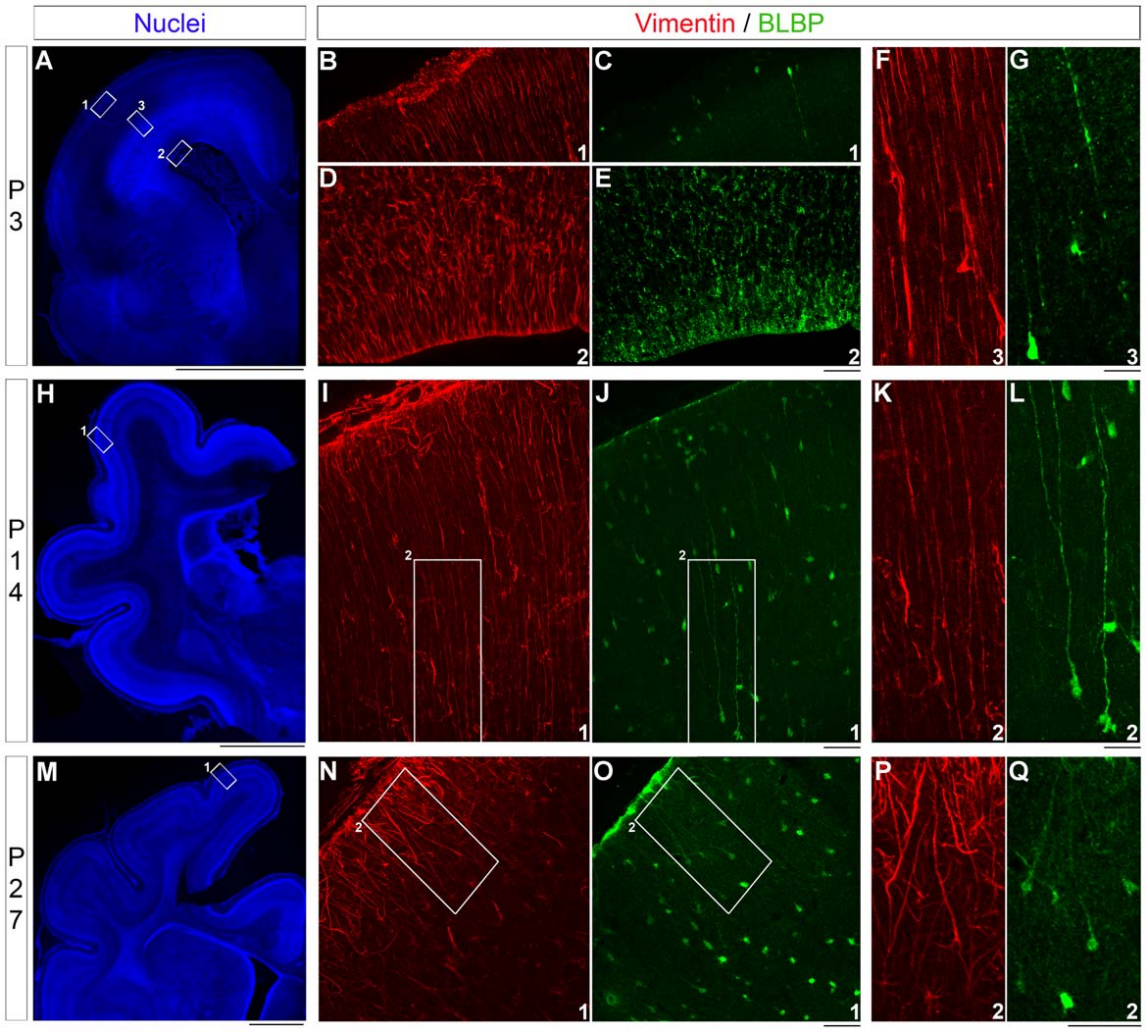
Expression of vimentin and BLBP during postnatal cortical development in normal ferrets. Immunostaining against vimentin (red) and BLBP (green) on coronal sections at P3 (**A–G**), P14 (**H–L**), and P27 (**M–Q**). Nuclear staining in blue with bisbenzimide (**A**,**H**,**M**). The boxed area in (**H**) and (**M**) are located in the somatosensory cortex. The same region is shown for vimentin immunoreactivity in red and BLBP immunoreactivity in green. (**F**) and (**G**) are higher magnification of radial glial processes at P3 within the cortical plate. The boxed area in (**I–O**) are shown at higher power in (**K–Q**). P: postnatal day. Vimentin expression is maintained in radial glial processes until P14 while BLBP expression is only expressed by a few radial glia at P3. At P14 and P27, BLBP labels radial glia in transition to astrocytes. Scale Bar: 50 µm (B–E,I,J,N,O), 100 µm (F,G,K,L,P,Q) and 2000 µm (A,H,M).

Vimentin is an excellent marker for radial glia in ferrets, but few others have been explored in this species [11], [14]. We also know that the radial glial phenotype differs among mammals. To further understand the relevant proteins/intermediate filaments expressed in ferret neocortex, we tested several other markers and observed that BLPB was strongly expressed. To expand our assessment of diversity among radial glia in normal ferret, we used immunostaining against both vimentin and BLBP during embryonic and postnatal development. Vimentin is expressed early, since it labels radial glial processes throughout the initial, mid, and final stages of corticogenesis in ferrets (E27, E33 and E39) (Figure 1). BLBP is also strongly expressed at E27 in the VZ, but not in radial processes (Figure 1C–E); whereas in the ganglionic eminence, radial processes express BLBP (Figure 1F). From E33 to E39 and P0, vimentin and BLBP colocolize in radial glial processes (Figure 1L–M,S–T). Elongated radial glia immunoreactive for vimentin are present from P3 to P14 (Figure 2F,K). At P3, BLBP immunoreactivity decreases in the VZ, and few BLBP+ cells are observed close to the pia (Figure 2C–E,G). These cells, also seen at P14, express vimentin and show an elongated process oriented toward the pia; interestingly, their cell bodies are in the cortical plate (Figure 2H,L). Four weeks after birth (P27), vimentin and BLBP label only radial glia in transition to astrocytes as shown here in the somatosensory cortex (Figure 2M–Q).

### Radial glial morphology is affected in MAM treated ferrets

**Figure 3:**
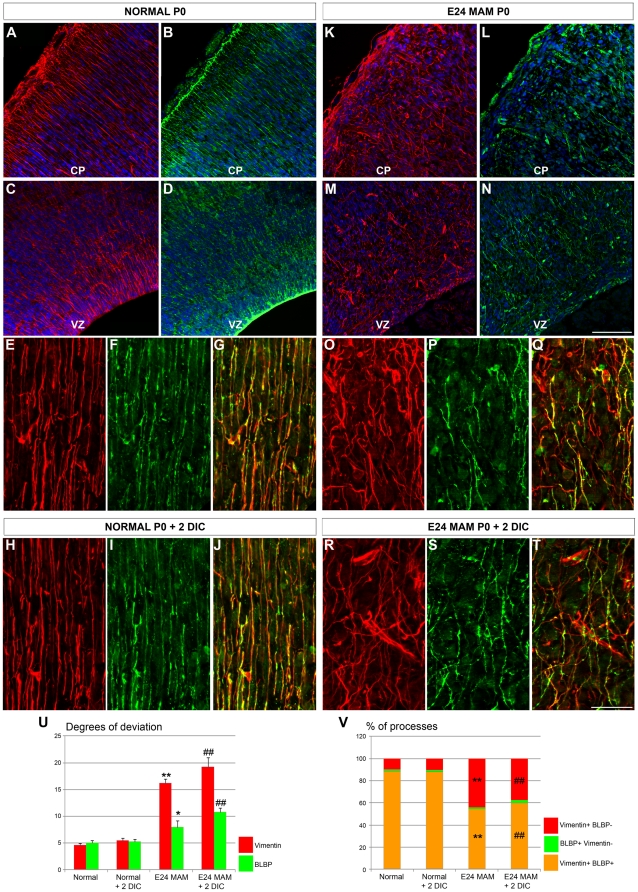
Morphology and phenotype of radial glial cells in normal and E24 MAM treated ferrets. Vimentin (red) and BLBP (green) immunostaining on coronal cortical sections cut on a cryostat (**A–N**) and organotypic slices maintained 2 DIC *in vitro* (**O–T**). Nuclear staining in blue with bisbenzimide. In normal ferrets, vimentin and BLBP expression is observed at the pial surface (**A–B**), in the ventricular zone (VZ) (**C–D**) and in elongated radial glial fibers in the cortical plate (CP) (**E–F**). Both markers colocolize as seen in the merged image (**G**). The same pattern of expression was maintained when normal slices were cultured for 2 days *in vitro* (**H–J**). In E24 MAM treated ferrets, the radial glial scaffold is severely disrupted (**K–Q**) and fewer vimentin-positive radial glial processes also express BLBP as seen in the merged picture (**Q**). Radial glial misalignment and BLBP downregulation were also observed in E24 MAM treated organotypic slices after 2 days *in vitro* (**R–T**). (**U**) is a graph of the degrees of deviation in radial glia. In normal ferrets (n = 6; 2 E38, 2 E39 and 2 P0), the low degree of deviation for vimentin+ and BLBP+ processes indicates that radial glia were relatively parallel. Similar results were obtained with normal ferrets slices maintained 2 days in culture (DIC) (n = 8; P0+2 DIC). The disrupted radial glial scaffold in MAM treated ferrets *in vivo* (n = 5; 3 E39 and 2 P0) and after 2 days *in vitro* (n = 6; P0+2 DIC) is illustrated by a large degree of deviation for vimentin+ processes. Although the lower degree of deviation for BLBP indicates that BLBP+ radial glia are only mildly disrupted compared with the vimentin+ cells in the MAM treated slices, they were significantly disrupted compared to BLBP+ cells in normal ferrets. (**V**) Histogram of the percentage of processes expressing vimentin and BLBP (vim+BLBP+, orange), only vimentin (vim+BLBP-, red) or only BLPB (BLBP+vim-, green). CP: Cortical Plate; VZ: Ventricular Zone. n =  number of slices; one slice/animal was analyzed. Error bars  =  standard error. Significance was determined using a Two-way ANOVA followed by pairwise multiple comparison procedures (Holm-Sidak method). *p = 0.013, **p≤0.001 compared to normal ferret; # = 0.001 compared to normal ferret +2 DIC. No statistical differences were found when we compared (i) normal ferrets *vs* normal ferrets +2 DIC and (ii) E24 MAM ferrets *vs* E24 MAM ferrets +2 DIC. Scale Bar: 25 µm.

Exposure to MAM at E24 leads to a severely disrupted radial glial scaffold [14], [21]. To assess whether all radial glial cells were disrupted, we compared the morphology of vimentin and BLBP immunoreactive radial glia in normal and MAM treated ferrets (E39-E40 or P0). These two markers and their colocalization were detected immunohistochemically in 20 µm thick sections or in organotypic slices maintained 2 DIC. As previously shown (Figure 1) in normal ferrets, vimentin+ and BLBP+ radial glia are elongated, parallel, and extend toward the pial surface, as opposed to an obviously disrupted appearance in E24 MAM-treated cortex (Figure 3A–T). To quantify radial glial morphology in normal and MAM treated brains, we calculated the degree of deviation of radial glial processes as described previously [21], [28], [39]. A low degree of deviation indicates that radial glia are elongated and parallel whereas a higher degree of deviation reveals disrupted and misaligned cells. Degrees of deviation are reported in Table 1. In normal ferrets, vimentin+ and BLBP+ radial glia display a low degree of deviation (Figure 3E–G,U). In MAM treated ferrets, a high degree of deviation occurs in vimentin+ radial glia (Figure 3O–Q,U). BLBP+ radial glia are substantially less disrupted after MAM treatment compared to the vimentin+ population (Figure 3E–F,O–P). Although the degree of deviation for BLBP+ radial glia is lower, it is significantly different from normal ferrets (p = 0.013; Figure 3F,I,P,S). The degree of deviation of radial glial processes (vimentin+ and BLBP+) observed in newborn ferrets is maintained after 2 days *in vitro* (in plain medium) compared with acute sections obtained from normal and E24 MAM treated ferrets (Figure 3H–T,U).

**Table 1:** Degrees of deviation of vimentin and BLBP+ radial glial processes in normal and E24 MAM treated *in vivo* and *in vitro* (2 DIC).

	Condition/Treatment	Vimentin	BLBB	n slices
**Fixed Brains**	Normal	4.63+/−0.31	4.99+/−0.50	6
	E24 MAM treated	16.16+/−0.79	7.98+/−1.19	5
**Normal Slices**	Plain medium	6.16+/−0.29	5.86+/−0.54	8
**E24 MAM treated Slices**	Plain medium	19.21+/−1.78	10.76+/−0.74	6
	Control HEK	18.10+/−1.33	12.02+/−1.05	7
	Recombinant Reelin	11.97+/−1.26	11.56+/−1.23	10
	Reelin HEK	10.74+/−0.56	10.29+/−0.49	6
	Reelin HEK+ RAP	20.19+/−0.45	12.89+/−0.19	7
	Reelin HEK+ SP600125	10.15+/−0.72	10.33+/−1.88	4
	Reelin HEK+ PP2	18.97+/−1.42	10.45+/−1.02	6
	Reelin HEK+ LY294002	18.02+/−0.94	12.49+/−0.73	9
	Reelin HEK+ TDZD-8	11.17+/−0.78	9.66+/−0.81	6
	Recombinant NRG1	10.43+/−0.89	9.22+/−1.81	4
	Ig-NRG1	11.08+/−0.53	9.72+/−1.04	4
	CRD-NRG1	20.51+/−1.29	10.97+/−1.03	4
	Rc NRG1 + erbB3 blocking antibodies	17.12+/−0.92	11.98+/−0.62	5
	Rc NRG1 + erbB4 blocking antibodies	20.02+/−2.62	14.30+/−1.24	6
	Rc NRG1 + LY294002	15.78+/−2.03	8.75+/−1.03	7

### Downregulation of BLBP in MAM treated ferret radial glial processes

Are vimentin and BLBP expression quantitatively changed? To answer this question, the proportion of radial glial processes single or double-labeled for vimentin and BLBP was determined on cryostat sections obtained from fixed brains or organotypic slices (maintained 2 days *in vitro*) in normal and MAM treated ferrets. These percentages are reported in Table 2. In normal ferrets, most of the vimentin+ processes are also BLBP+ (88.79%); only 10.02% of vimentin+ processes do not express BLBP (Figure 3E–G,V). In MAM treated ferrets however, only 54.45% of radial glial processes express both markers and 44.15% express only vimentin (Figure 3O–Q,V). After 2 DIC, the percentage of processes expressing both vimentin and BLBP is higher in normal than in MAM treated slices (Figure 3H–J,R–T,V). In contrast to normal ferrets where both markers colocalize, two distinct populations of radial glia exist in MAM treated ferrets: radial glial cells expressing only vimentin are strongly disrupted whereas radial glial cells expressing both markers (vimentin+BLBP+ radial glial cells) are only mildly disrupted. These characteristics are maintained after 2 days *in vitro* (Figure 3E–J,O–T,V).

**Table 2:** Proportion of radial glial processes expressing vimentin, BLBP or both in normal and E24 MAM treated *in vivo* and *in vitro* (2 DIC).

	Condition/Treatment	Vimentin+ BLBP+	Vimentin-BLBP+	Vimentin+ BLBP-	n slices
**Fixed Brains**	Normal	88.79+/−0.72	1.17+/−0.17	10.02+/−0.74	6
	E24 MAM treated	54.45+/−3.38	1.38+/−0.27	44.15+/−3.51	5
**Normal Slices**	Plain medium	87.98+/−0.57	1.70+/−0.19	10.31+/−0.41	8
**E24 MAM treated Slices**	Plain medium	60.11+/−3.4	2.17+/−0.89	37.71+/−3.99	6
	Control HEK	61.73+/−3.29	2.26+/−0.76	35.99+/−3.78	7
	Recombinant Reelin	72.56+/−1.83	2.46+/−0.86	24.97+/−1.67	10
	Reelin HEK	71.81+/−1.68	1.64+/−0.80	26.53+/−2.04	6
	Reelin HEK+ RAP	65.56+/−2.49	2.93+/−0.65	31.50+/−2.50	7
	Reelin HEK+ SP600125	73.92+/−3.35	0.97+/−0.34	25.10+/−3.02	4
	Reelin HEK+ PP2	65.56+/−2.49	2.93+/−0.65	31.50+/−2.50	6
	Reelin HEK+ LY294002	65.41+/−2.45	1.26+/−0.54	33.32+/−2.76	9
	Reelin HEK+ TDZD-8	73.01+/−1.53	0.77+/−0.49	26.20+/−1.58	6
	Recombinant NRG1	68.98+/−2.13	2.13+/−0.39	28.88+/−2.54	4
	Ig-NRG1	69.75+/−0.54	1.94+/−0.64	28.29+/−0.89	4
	CRD-NRG1	58.74+/−4.04	1.84+/−0.71	39.40+/−4.12	4
	Rc NRG1 + erbB3 blocking antibodies	61.04+/−2.03	1.49+/−0.54	37.46+/−1.66	5
	Rc NRG1 + erbB4 blocking antibodies	59.76+/−4.04	2.26+/−0.32	37.96+/−0.77	6
	Rc NRG1 + LY294002	62.21+/−1.92	3.10+/−0.65	34.67+/−1.89	7

### The full length or the central fragment of reelin realigns disrupted radial glia *via* VLDLR/Dab1/Pi3K activation

**Figure 4:**
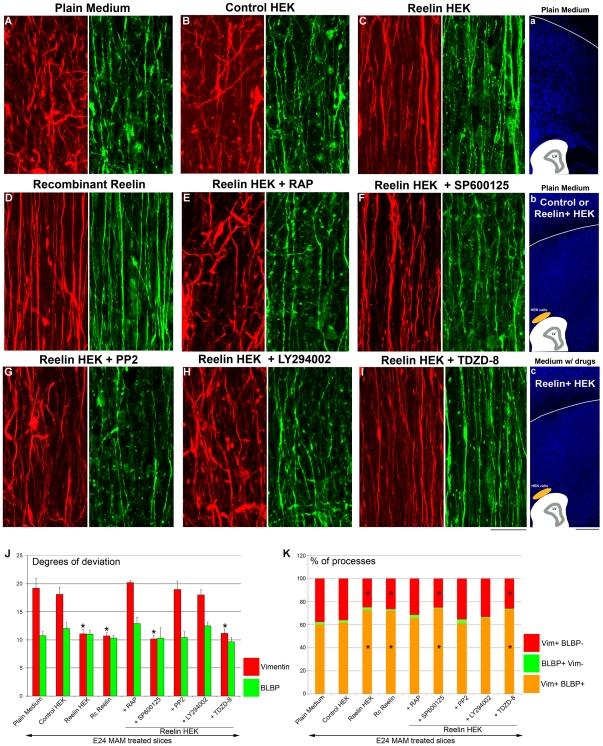
Deviation of vimentin+ and BLBP+ fibers in E24 MAM treated organotypic cultures exposed to reelin. (**a–c**) on the right side of the figure are bisbenzimide stained images of organotypic cultures maintained under different conditions. On the bottom of each image is a diagram of the slice and associated coculture with HEK cells. (**A–I**) Immunostaining against vimentin (red) and BLBP (green) after 2 days in culture (DIC). In control experiments, E24 MAM slices were cultured in plain medium (**A**, n = 6; [**a**]) or cocultured with control HEK cells (**B**, n = 7) included in Matrigel and placed at the pial surface as shown in (**b**). The radial glia remain disordered in these control conditions. To assess the role of reelin, organotypic slices were cocultured with HEK cells secreting reelin (**C**, n = 6) included in Matrigel and placed at the pial surface (as shown in [**c**]), or cultured in a medium containing the central fragment of reelin (**D**, n = 10). Some slices cocultured with reelin+ HEK cells were also incubated in a medium supplemented with the following drugs: 300 nM RAP (**E**, n = 7), or 10 µM SP600125 (**F**, n = 4), or 10 µM PP2 (**G**, n = 6), or 50 µM LY294002 (**H**, n = 9) or 56 µM TDZD-8 (**I**, n = 6). (See Figure S1). (**J**) Histogram of the degrees of deviation for vimentin+ and BLBP+ radial glial processes. (**K**) Histogram of the percentage of processes expressing vimentin and BLBP (vim+BLBP+, orange), only vimentin (vim+BLBP-, red), or only BLPB (BLBP+ vim-, green). An increase of the percentage of radial glial processes expressing vimentin and BLBP is correlated with repair of radial glial morphology (lower degrees of deviation). This effect induced by reelin is mediated *via* VLDLR/Dab1/Pi3K activation. n =  number of slices. Error bars  =  standard error. Significance was determined using a Two-way ANOVA followed by pairwise multiple comparison procedures (Holm-Sidak method). Significant pairwise comparisons are between control (*i.e.* Plain Medium) and tested conditions. *p≤0.002. Scale Bar: 25 µm (A–I) and 500 µm (1–3).

In MAM treated brains, Cajal-Retzius cells expressing reelin are highly disorganized [21]. Exogenous reelin placed at the pial surface, however, realigns disrupted radial glia [39]. To test whether cells expressing vimentin and/or BLBP respond differently to repair signals, MAM treated organotypic slices were incubated for 48 h in the presence of reelin. After culture, we visualized radial glia by immunodetection for vimentin and BLBP; the degrees of deviation were calculated (see Table 1). To create a focal and normotopic source of reelin, MAM slices were co-cultured with reelin-secreting HEK cells included in Matrigel and placed at the pial surface [39] (Figure 4a–c). To assess the effect of a diffuse source of reelin, MAM treated slices were incubated in medium containing recombinant reelin (1 nM) or plain medium as a control. Only vimentin+ radial glia were changed when slices were cocultured with reelin+ HEK cells or recombinant reelin; Figure 4C–D,J) compared to controls (control HEK cells and plain medium, Figure 4A–B,J). In contrast, the degree of deviation of BLBP+ radial glia did not alter compared to controls (Figure 4J). Therefore exogenous reelin improves the morphology of vimentin+ radial glial processes, which are also the most disrupted; but did not modify the mildly disrupted morphology of BLBP+ cells. Another interesting finding is that the highly polarized morphology of radial glia is restored whether the source of reelin is focal or diffuse. To further clarify the mechanism of radial glial repair induced by reelin, the morphology was analyzed in MAM treated slices cocultured with reelin+ HEK cells in media supplemented with drugs that influence reelin signaling (Figure S1). RAP (300 nM) (human recombinant Receptor Associated Protein), prevents the binding of reelin to both ApoE2R and VLDLR [46], [47]. This drug eliminated the radializing effect of reelin, suggesting that activation of ApoER2 and VLDL receptors is necessary (Figure 4E,J). ApoER2 and VLDLR exhibit overlapping but also distinct functions in the transduction of the reelin signaling [48]. Their activation also recruits different intracellular signaling cascades. ApoER2, unlike VLDLR, recruits two intracellular proteins, JNK-interacting proteins-1 and 2, JIP-1 and-2 [49]. We took advantage of this difference to evaluate the role of ApoER2. We inhibited JIP activity by applying SP600125 (50 µM). This drug does not prevent the effect mediated by simultaneous administration of reelin suggesting that ApoER2 activation is not required to repair the morphology of vimentin+ radial glia (Figure 4F,J). To further assess the influence of intracellular reelin signaling, we used PP2 (10 µM), a Src inhibitor, which blocks Dab1 phosphorylation, as well as LY294002, which by inhibiting PI3K prevents the activation of Akt. Blockade of either Dab1 or Pi3K resulted in continued severe disruption of vimentin+ processes in the presence of reelin whereas BLBP+ processes were unchanged compared to controls (slices cultured in plain medium or cocultured with control HEK cells) (Figure 4G–H,J). Finally we used TDZD-8 (56 µM) to block GsK3β, a target of Akt known to be involved in the reelin signaling [50]. In this condition, the exogenous reelin resulted in improved radial glial morphology (Figure 4I,J).

### Reelin affects the number of radial glial processes expressing BLBP *via* VLDLR/Dab1/Pi3K activation

The percent of radial glial processes expressing vimentin and BLBP is decreased in MAM treated cortex compared to normal (Figure 3V). To test whether exogenous reelin also affects BLBP expression, the number of processes expressing both radial glial markers, vimentin and BLBP, or only one these markers, was quantified (see Table 2). As in control conditions, slices were incubated in plain medium or cocultured with control HEK cells, which also showed a reduced number of radial glial processes expressing BLBP (Figure 4A–B,K). In the presence of reelin (recombinant reelin or reelin secreted by HEK cells), the number of processes expressing both markers, vimentin and BLBP, increases (Figure 4C–D,K). When slices are cocultured with reelin+ HEK cells in presence of: RAP, PP2, and LY294002, the percentage of radial glial processes expressing both markers, vimentin and BLBP, does not increase and is similar to controls (*i.e.*, E24 MAM treated slices in plain medium or control HEK cells; Figure 4E,G,H,K). Finally, blocking ApoER2, or GsK3β does not prevent the effect induced by reelin (Figure 4F,I,K).

### Activation of ApoER2, Dab1, and PI3K but not VLDLR and GSK3β is required for the reelin-mediated effect on neuronal migration

**Figure 5:**
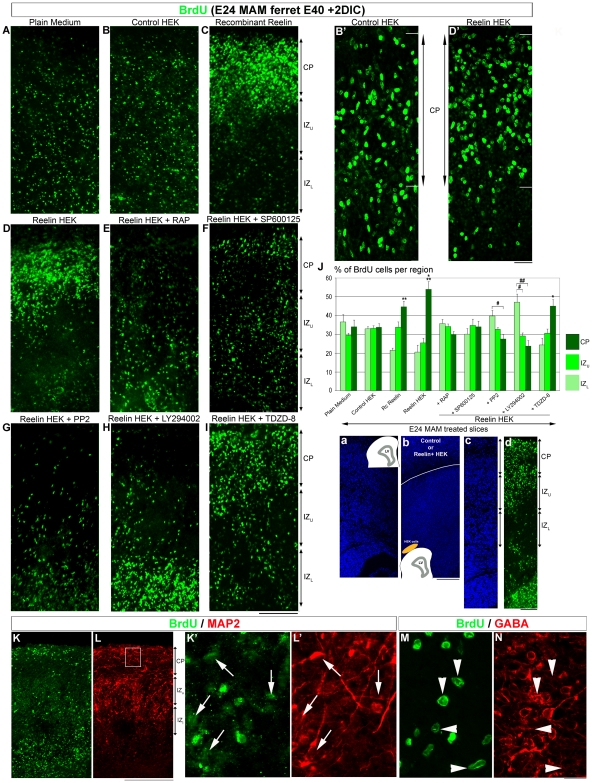
Position of BrdU+ cells in E24 MAM treated organotypic cultures exposed to reelin. In the middle right of the figure (**a–b**) are bisbenzimide stained images of organotypic cultures maintained under different conditions. In each image is a diagram of the slice and associated coculture with HEK cells. (**c–d**) show the zones analyzed for the position of BrdU+ cells after 2 days in culture (DIC) (**c**) is bisbenzimide staining and (**d**) is BrdU immunostaining after coculture with reelin+ HEK cells. After a pulse of BrdU, MAM treated slices were incubated for 2 DIC in plain medium (**A**, n = 6) as shown in (**a**), or cocultured with control HEK cells (**B**, n = 8) as seen in (**b**); HEK cells included in Matrigel and placed at the pial surface are shown in yellow (**b**). Some slices were incubated with recombinant reelin (**C**, n = 8) or cocultured with reelin secreting HEK cells (**D**, n = 6 as shown in [**b**]). To inhibit specific steps of the reelin pathway, other slices were cocultured with HEK cells secreting reelin in a medium supplemented with 300 nM RAP (**E**, n = 8), 10 µM SP600125 (**F**, n = 11), 10 µM PP2 (**G**, n = 5), 50 µM LY294002 (**H**, n = 6), or 56 µM TDZD-8 (**I**, n = 5). (See Figure S1). (**B'**) shows a high magnification of BrdU+ cells in the cortical plate (CP) in slices cocultured with control HEK cells while (**D'**) shows an image of BrdU immunoreactivity in an organotypic slice cultured with reelin+ HEK cells. (**J**) is a histogram indicating the distribution of BrdU+ cells after different treatments. (**K–N**) Organotypic MAM treated slice (E40) incubated 2 DIC with recombinant reelin (1nM), resectioned using a cryostat at 14 µM. Double immunostaining for BrdU (**K**, green) and MAP2 (**L**, red). (**K'–L'**) Higher magnification of the boxed area in **L**. The arrows indicate BrdU-positive cells that are also MAP2-positive. Double immunostaining for BrdU (**M**, green) and GABA (**N**, red). The arrow heads indicate BrdU+ cells that are GABA-negative. CP: Cortical Plate; IZ_u-L_: Upper and Lower Intermediate Zone. n =  number of slices. Error bars  =  standard error. Significance was determined using a Two-way ANOVA followed by pairwise multiple comparison procedures (Holm-Sidak method). *p = 0.017, **p = 0.003, ***p = 0.001 compared to CP in control medium. #p = 0.017, ##p = 0.025 compared to IZ_L_. Scale Bar: 50 µm (A–I), 25 µm (K',L',M,N), 250 µm (K–L), and 500 µm (a–d).

Reelin not only repairs the radial glial scaffold but also facilitates the migration of neurons into the CP [39]. To understand the process induced by reelin, MAM treated organotypic slices were exposed to a pulse of BrdU (1 h) and incubated with reelin (reelin secreted by HEK cells or recombinant reelin) in the presence of drugs blocking specific steps of the reelin pathway. After 2 DIC, the positions of BrdU+ cells were analyzed in 3 different cortical regions: the cortical plate (CP), the upper (IZ_U_) and lower (IZ_L_) parts of the intermediate zone (as described in [39]) (Figure 5c–d). Results are reported in Table 3. In MAM treated slices incubated in plain medium or cocultured with control HEK cells, BrdU+ cells tend to scatter in all cortical layers (Figure 5A,B,J). However, in the presence of either the central fragment (recombinant reelin) or the full length reelin (reelin secreting HEK cells), BrdU+ cells strongly accumulate in the CP (Figure 5C,D,J). The majority of BrdU+ cells found in the CP, after 2 DIC, are also likely to be generated in the neocortical VZ since they express MAP2 but are GABA-negative (Figure 5K–N). Also when DiI crystals are placed in the ganglionic eminence of MAM treated slices, few DiI+ cells were found in the cortex after 2 DIC; about 3–4 days are needed for tangentially migrating cells to reach the neocortex [22]. No statistical differences in the distribution of BrdU+ cells were found when MAM slices were cocultured with reelin HEK cells in medium containing RAP or SP600125, suggesting that neuronal migration is mediated mainly by ApoER2, with little if any contribution from VLDLR (Figure 5E,F,J). By using pharmacologic blockade of specific elements of the reelin signaling pathway, we observed that accumulation of cells in the CP induced by exogenous reelin secreted by HEK cells required the activation of Dab1 (Figure 5G,J), and Pi3K (Figure 5H,J). After blockade of Dab1 with PP2, fewer cells reached the CP and many settled near the ventricular zone, only able to travel short distances. Inhibiting Pi3K had a greater effect in this distribution, suggesting that reelin is not the only signal needed to initiate neuronal migration. Finally, blocking GSK3β, an element downstream of Pi3K, did not prevent the effect induced by reelin since many BrdU+ cells are found in the CP (Figure 5I,J). Our data suggest that (1) reelin stimulates migration toward the CP *via* activation of ApoER2, in a Dab1- and Pi3K-dependent but VLDLR- and GSK3β-independent manner and (2) reelin-dependent signaling as well as reelin-independent but Pi3K-dependent signaling facilitate neuronal migration from lower IZ toward the CP (see Figure S1 and also Figure 8).

**Table 3:** Distribution of BrdU+ cells in the cortical plate (CP), the upper IZ (IZ_u_) and in the lower IZ (IZ_L_).

Condition/Treatment	CP	IZ upper	IZ lower	n slices
Plain medium	33.98+/−3.61	29.46+/−1.15	36.54+/−4.08	6
Control HEK	33.83+/−1.78	33.18+/−1.35	32.97+/−1.10	8
Recombinant Reelin	44.68+/−3.02	33.81+/−2.81	21.50+/−1.26	8
Reelin HEK	53.98+/−4.08	25.46+/−2.50	20.54+/−3.77	6
Reelin HEK+ RAP	29.98+/−1.48	34.21+/−1.34	35.79+/−2.17	8
Reelin HEK+ SP600125	34.06+/−2.93	34.78+/−3.35	30.07+/−3.07	11
Reelin HEK+ PP2	27.59+/−2.35	32.67+/−0.98	39.72+/−3.01	5
Reelin HEK+ LY294002	23.78+/−2.97	29.14+/−1.62	47.06+/−4.39	6
Reelin HEK+ TDZD-8	45.09+/−3.48	30.55+/−2.31	24.35+/−3.66	5
Recombinant NRG1 (1 nM)	30.31+/−2.88	35.30+/−0.81	34.37+/−2.85	5
Recombinant NRG1 (30 nM)	32.66+/−1.67	32.28+/−1.19	35.05+/−0.65	4
Ig-NRG1	35.15+/−1.78	30.47+/−1.96	34.37+/−1.10	7
CRD-NRG1	34.67+/−2.29	32.23+/−1.33	33.09+/−1.67	6
Forskolin	31.80+/−1.89	36.07+/−2.59	32.11+/−1.47	3
Recombinant NRG1 + Forskolin	33.22+/−1.63	32.87+/−0.87	33.90+/−0.75	3

Organotypic slices were obtained from E24 MAM treated ferrets and maintained *in vitro* (2 DIC).

### Radial glial morphology is improved in the presence of an exogenous secreted form of NRG1

**Figure 6:**
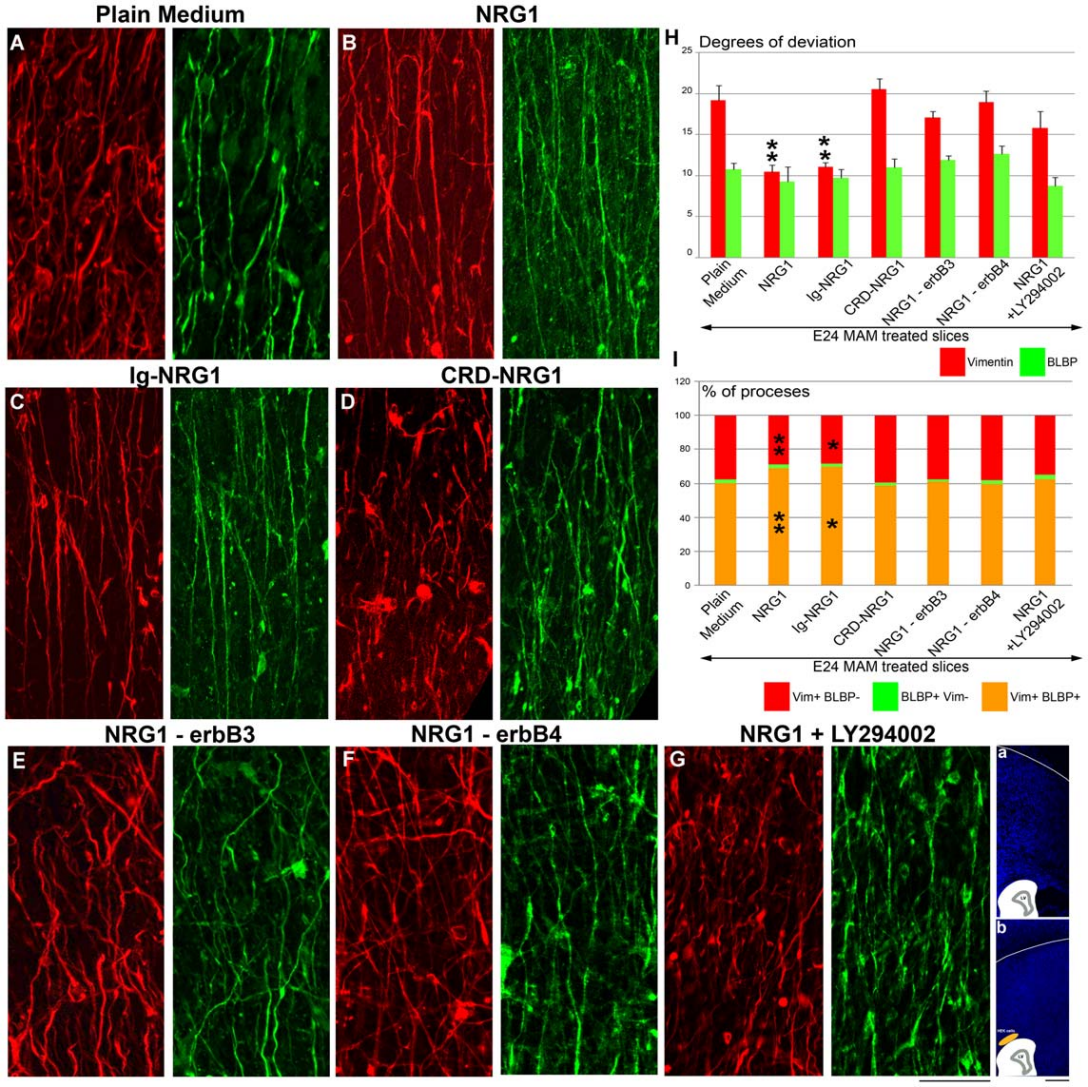
Deviation of vimentin+ and BLBP+ fibers in E24 MAM treated organotypic cultures exposed to variant forms of NRG1. (**A–G**) Immunostaining against vimentin (red) and BLBP (green) after 2 DIC. (**A**, n = 6), depicts control slices incubated in plain medium. Vimentin+ radial glia realign when MAM treated slices are incubated with 1 nM of recombinant NRG1 (**B**, n = 4) or cocultured with Ig-NRG1 cells (**C**, n = 4). The morphology of vimentin+ radial glia was not improved in cocultures with CRD-NRG1 cells (**D**, n = 4). The effect of recombinant NRG1 was abolished in presence of antibodies blocking erbB3 (20 µg/ml) (**E**, n = 5) or erbB4 (20 µg/ml) (**F**, n = 6), and in presence of a Pi3K inhibitor LY294002 (50 µM) (**G**, n = 7). (See Figure S1). (**a**) illustrates slices in **A–B**,**E–F** cultured in plain medium or medium supplemented with drugs. Slices in **C** and **D** were cocultured with HEK cells as shown in (**b**). (**H**) Histogram illustrating the degrees of deviation for vimentin+ and BLBP+ radial glial processes. (**I**) Histogram of the percentage of processes expressing vimentin and BLBP (vim+BLBP+, orange), only vimentin (vim+BLBP-, red) or only BLPB (BLBP+ vim-, green). n =  number of slices. Error bars  =  standard error. Significance was determined using a Two-way ANOVA followed by pairwise multiple comparison procedures (Holm-Sidak method). **p<0.001, *p = 0.003. Scale Bar: 25 µm.

Soluble recombinant type I NRG1 repairs the radial glial scaffold in E24 MAM ferret slices [28]. To assess whether NRG1 acts similarly on both vimentin+ and BLBP+ radial glia, organotypic slices were exposed for 2 DIC to NRG1, and the degrees of deviation computed for vimentin+ and BLBP+ radial glial processes (see Table 1). MAM treated slices were initially exposed to a diffuse source of recombinant NRG1 (1 nM) as described previously [28]. We observed a dramatic realignment of vimentin+ radial glia with an angle of deviation at 10.43, which is significantly reduced from the angle of deviation of vimentin+ radial glia in MAM treated slices incubated 2 DIC in plain medium (19.21) (Figure 6A,B,H). As described above using reelin, BLBP+ radial glial morphology, although much less disrupted in MAM treated ferrets compared to vimentin+ radial glia, was unchanged after treatment with recombinant NRG1 (9.22 compared to 10.76 in plain medium; not significant, p>0.05). Again, the morphology of BLBP+ radial glia was similar to the improved vimentin+ processes. The soluble recombinant NRG1 used here is a truncated form of NRG1, which contains only the soluble EGF-like domain. This form of NRG1, commonly used for *in vitro* studies, is sufficient to elicit ErbB receptor dimerization, tyrosine phosphorylation and the activation of downstream signaling pathways [51]. We additionally decided to expose MAM treated slices to the full length of NRG1 to assess if other domains beyond the EGF-like domain could improve the BLBP+ radial glial morphology. E24 MAM slices were co-cultured with HEK cells secreting the full length of type I NRG1 (Ig-NRG1 cells). HEK cells were included in Matrigel and placed at the pial surface as described previously. The morphology of vimentin+ radial glial cells was dramatically improved, comparable to a treatment with soluble recombinant NRG1 (degree of deviation of 11.08; Figure 6C,H). However, BLBP+ radial glia remained the same in the presence of the full length of NRG1 (degree of deviation of 9.72), but similar to the improved vimentin+ radial glia. Finally, we co-cultured MAM slices with HEK cells expressing type III NRG1 (CRD-NRG1 cells). The isoform III, unlike the Ig-like domain of type I NRG1, is not secreted and contains a cysteine-rich domain (CRD). We found no improvement of the radial glial scaffold: with a degree of deviation of 20.51 for vimentin and 10.91 for BLBP, suggesting that the morphology of radial glia in presence of type III NRG1 was similar to MAM slices cultured in plain medium (Figure 6D,H). Therefore, the soluble EGF-like domain of type I NRG1, applied diffusely or focally, is sufficient to realign vimentin+ radial glia, which are highly disrupted in MAM treated animals; BLBP+ radial glia, although less disrupted, remain unchanged.

### Blockade of erbB3/erbB4 or inhibition of Pi3K signaling prevents the realignment of radial glia induced by NRG1

To further elucidate the mechanism of radial glial radialization induced by NRG1, MAM slices were cultured in medium containing recombinant NRG1 (1 nM) combined with HER-3 Ab-5 or HER-4 Ab-3, two antibodies blocking the binding of NRG1 to erbB3 and erbB4 respectively (Figure S1). Radialization of vimentin+ radial glia mediated by NRG1 was prevented by blocking erbB3 or erbB4 receptors (Figure 6E,F,H). The morphology of BLBP+ processes however demonstrated only slight changes, which were not significant. Since erbB receptors activate the Akt/Pi3K signaling pathway (for review see [52]), slices were cultured with recombinant NRG1 combined with LY294002 (50 µM), a Pi3K inhibitor. In these conditions, the effect of NRG1 on vimentin+ radial glia was significantly reduced when Pi3K was inhibited. Blocking Pi3K does not alter the morphology of BLBP+ radial glia (Figure 6G,H). Together, these results indicate that NRG1 *via* activation of erbB3/erbB4 receptors and Pi3K induces a realignment of vimentin+ radial glia (see also Figure 8).

### Increased expression of BLBP in the presence of NRG1 is erbB3/4 and Pi3K dependent

The number of radial glial processes co-expressing vimentin and BLBP was significantly increased compared to control when slices were incubated in the presence of NRG1 or cocultured with Ig-NRG1 cells (Figure 6I, Table 2). No significant difference was found when slices were cocultured with CRD-NRG1 cells compared to MAM treated slices incubated in plain medium (control experiment). The percent of vimentin+ radial glial processes expressing BLBP in presence of NRG1 while blocking erbB3, erbB4 or Pi3K was similar to control (Figure 6I, Table 2).

### NRG1 does not improve neuronal migration toward the cortical plate

**Figure 7:**
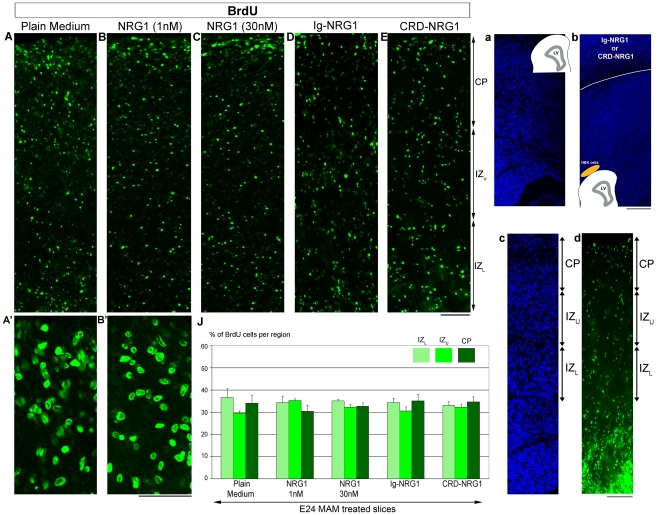
Position of BrdU+ cells in E24 MAM treated organotypic cultures exposed to variant form of NRG1. After a pulse of BrdU, MAM treated slices were incubated 2 days in culture (DIC) in plain medium (**A**, n = 6) or in a medium containing 1 nM (**B**, n = 5) or 30 nM (**C**, n = 4) of recombinant NRG1 as shown in (**a**). Some slices were also cocultured with Ig-NRG1 HEK cells (**D**, n = 7) or CRD-NRG1 HEK cells (**E**, n = 6) as shown in (**b**). High magnification of BrdU+ cells in the cortical plate (CP) after 2 DIC in plain medium (**A'**) or in presence of 1 nM NRG1 (**B'**). The positions of BrdU+ cells in 3 cortical compartments, CP, upper and lower intermediate zone (IZ_U_ and IZ_L_) was analyzed after 2 DIC as shown in (**c**) (nuclear staining) and (**d**) (BrdU immunostaining of a slice cultured in plain medium). (**J**) Histogram of the position of BrdU+ cells. No significant differences were found between control (plain medium) and different forms of NRG1; in all conditions, BrdU+ cells distribute in a typical pattern for an E24 MAM treated slice in that they are spread throughout cortical wall. n =  number of slices. Error bars  =  standard error. Two-way ANOVA followed by pairwise multiple comparison procedures (Holm-Sidak method). Scale Bar: 150 µm.

**Figure 8:**
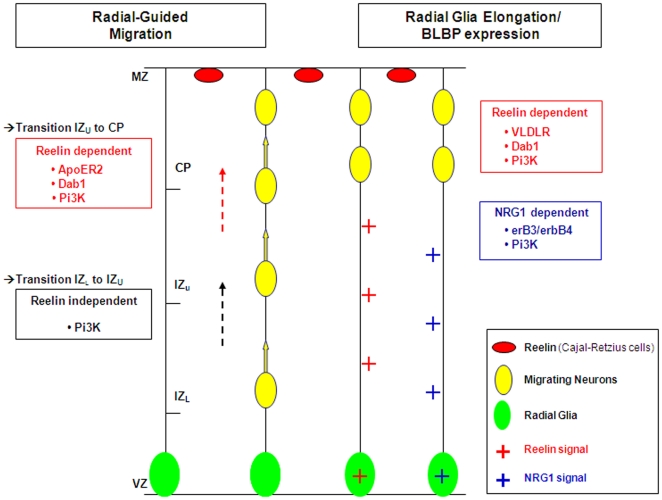
Schematic view of the role of reelin and NRG1 during late corticogenesis in ferret. Reelin secreted by Cajal-Retzius cells assists the migration of neurons from the upper intermediate zone (IZ) toward the cortical plate (CP). This process requires the activation of ApoER2, Dab1 and Pi3K. The transition from lower IZ to upper IZ is reelin-independent but Pi3K-dependent. Radial glial elongation is also influenced by reelin *via* activation of VLDLR, Dab1 and Pi3K. NRG1 does not control radial-guided migration in ferret but controls radial glial elongation *via* activation of erbB3, erbB4 and Pi3K. BLBP expression in radial glial processes is correlated with their elongated morphology and can be upregulated by reelin and NRG1. CP: Cortical Plate; IZ_u-L_: Upper and Lower Intermediate Zone; MZ: Marginal Zone.

In MAM treated ferrets, disruption of radial glia is associated with abnormal migration; neurons are scattered in all cortical layers compared to normal ferrets where neurons accumulate in an inside-out pattern [21]. Since NRG1 can repair the radial glial scaffold [28], we tested whether neuronal migration was also restored. Slices were exposed to a pulse of BrdU+ and the distribution of BrdU+ cells was evaluated after 2 DIC as described in Figure 4 (see also Figure 7c–d and Table 3 for details). Slices were incubated in plain medium or in medium containing 1 nM of soluble recombinant NRG1 or a 30 nM concentration, which has been shown to promote migration along the radial glial scaffold in mice [23]. In the presence of NRG1 (1 nM or 30 nM), BrdU+ cells scattered in the cortical wall, similar to control conditions (slices incubated in plain medium) (Figure 7A–C,J). We also used forskolin, a drug known to enhance the level of NRG1 receptors available at the membrane, *via* an increase of intracellular cyclic AMP [53]. Slices were treated with forskolin alone (2 µM) or forskolin (2 µM) combined with NRG1 (1 nM). Forskolin treatment left the distribution of BrdU+ cells unchanged suggesting that failure to migrate into the CP is not due to decreased receptors at the membrane (Table 3). To test whether the full length type I NRG1 or membrane bound type III NRG1 could improve radial migration, MAM slices were co-cultured with Ig-NRG1 or CRD-NRG1 HEK cells. Figure 7D,E,J show that BrdU+ cells failed to accumulate in the CP in both conditions. Migration into the CP was not improved in any of the conditions supplying exogenous NRG1: recombinant NRG1 (1 nM w/o forskoline, or 30 nM), co-culture with cell lines expressing the secreted form of type I NRG1 (Ig-NRG1 cells) or the membrane form of type III NRG1 (CRD-NRG1 cells). This suggests that a normal radial glial scaffold is not sufficient to restore neuronal migration and other factor(s) are essential to direct migrating neurons toward the CP (see also Figure 8).

## Discussion

We show here that severely disrupted dyplasic brains can be repaired by specific application of reelin or NRG1. In our model of cortical dysplasia, reelin restores the radial glial scaffold as well as glia-guided migration; NRG1, however, had a more limited effect since radial glia were realigned, but neuronal migration was not improved (Figure 8). The differential expression of BLBP and vimentin during normal and abnormal cortical development suggest a key role of BLBP in radial glial elongation and possibly in protection against environmental insults.

### VLDLR and ApoER2 exert different functions during cortical development

Corticogenesis in E24 MAM treated ferrets is severely disrupted; misaligned radial glial cells differentiate into astrocytes and neurons fail to reach the cortical plate [14], [21], [22]. Reelin is not missing but ectopic Cajal-Retzius cells most likely provide aberrant signaling due to their abnormal placement [14], [21], [39]. We previously demonstrated that an exogenous source of reelin placed at the pial surface was sufficient to restore the radial glial scaffold and neuronal migration toward the cortical plate [39]. Here we demonstrate that these effects are Dab1- and Pi3K- dependent but possibly mediated through different receptors. The role of the reelin receptors ApoER2 and VLVDR was evaluated using RAP, which blocks both receptors [46], [47], or using SP600125 which blocks ApoER2 by inhibiting JIP activity [49]. We found that during late corticogenesis glia-guided migration is influenced by ApoER2 as demonstrated previously in mice by Hack et al. [48] RAP prevents the repair of radial gial cells induced by reelin whereas SP600125 had no effect. This result suggests that reelin signaling is mediated mainly by VLDLR during late corticogenesis with little if any contribution from ApoER2. This finding may be mitigated by the observation that ApoER2 is expressed in stem cells, while JIP-1 and JIP-2 are not. Radial glia, of course, are a form of stem cells, therefore the results of Stockinger et al. [49] suggest that JIP-1 and JIP-2 are expressed in neurons but not in radial glial cells. However, a population of stem cells produced from mouse cortical cells of 15–17 old day embryos and grown in the presence of growth factors may not express the same set of factors/genes produced by newborn ferret radial glia *in vivo* or grown in organotypic cultures. In addition, Hack et al. [48] reported that radial glial morphology is not affected in ApoER2-/- mice, strongly supporting the idea that ApoER2 is not necessary for maintaining radial glial elongation.

Others report that the N-terminal portion of reelin binds to cadherin-related neuronal receptors and the integrin receptors [54], [55]. Jossin et al. [56] demonstrate that the central fragment of reelin binds to ApoER2 and VLDLR but does not bind to cadherin-related neuronal receptors. We found no differences when slices were treated with recombinant reelin, which consists of the central fragment of reelin, or when slices were cocultured with HEK cells secreting the full length of reelin. Since the central fragment of reelin is sufficient to both realign the radial glial scaffold and improve neuronal migration, it is likely that neither the integrin receptors nor the cadherin-related neuronal receptors are essential for migration in our model.

### Reelin is essential to glia-guided migration in gyrencephalic cortex

Reelin not only repairs radial glia but also improves migration of neurons that are likely to be generated in the neocortical ventricular zone since they express MAP2 and are also GABA-negative. In the presence of exogenous reelin, neurons move into the cortical plate but only into the lower intermediate zone when Dab1 and Pi3K were inhibited, suggesting that reelin signaling is essential for neuronal migration from the intermediate zone toward the cortical plate. This is supported by Uchida et al. [57] who demonstrated that radially migrating neurons in the subventricular/intermediate zone strongly express functional VLDLR and ApoER2 receptors, which then downregulate in the cortical plate. We found that interfering with Pi3K function produced more cells accumulating in the lower intermediate zone than blocking Dab1, indicating that in addition to reelin, another unknown Pi3K-dependent signaling pathway is also involved as suggested by Jossin and Goffinet (2007) [58]. Another possibility is that blockade of Pi3K exhibits a more severe phenotype due to the pleiotropic functions controlled by PI3K/Akt signaling [59]. Jossin and Goffinet (2007) proposed that inhibition of Pi3K impairs the polarity of neurons so they accumulate in the intermediate zone. Morphological transition from multipolar to bipolar neurons is essential to reach the cortical plate [60], [61]. In birds, the pallium develops in an outside-inside gradient and migrating neurons display a multipolar morphology. Nomura et al. [62] found that avian migrating neurons adopt a bipolar shape when reelin signaling is experimentally increased. These studies, as well as our observations, clearly indicate that reelin by its duel function on radial glia and neuronal migration is fundamental to the development of 6-layered lissencephalic as well as gyrencephalic cortices.

### NRG1 repairs the radial glial scaffold but not radial migration

NRG1 signaling is also essential for the normal development of radial glia [23], [63], [28]. In E24 MAM treated ferrets, NRG1 is reduced and treatment with recombinant NRG1 realigns radial glial morphology [28]. We demonstrate here that NRG1 realigns vimentin-positive but does not significantly alter BLBP-positive radial glia. We also show that the effect mediated by NRG1 is erbB3/erbB4 and Pi3K-dependent. This is consistent with previous reports showing that erbB3 and erbB4 are expressed by radial glia [23] and mediate radial glial elongation [23], [63], [28]. We also found that, unlike reelin, NRG1 does not improve radial guided migration. Anton et al. [23] show that neurons in the cortical plate express erbB receptors and that NRG1 stimulates migration along radial glia. Others report that interneurons born in the ganglionic eminences express erbB4 and tangential migration toward the dorsal telencephalon is partially controlled by NRG1 *via* erbB4 [64], [65]. This is consistent with our previous observations that NRG1 improves the radial phase of interneuron migration toward the cortical plate in E24 MAM treated slices [22].

Time-lapse imaging studies revealed that in postnatal ferret visual cortex translocating neurons with a long pial-contacting process coexist with short-process locomoting neurons [66]. In mice however, translocation is observed at the early stages when the cortical wall is relatively thin whereas locomotion is more abundant in late corticogenesis [67], [68], [69]. This difference between mouse and ferret suggest that signals controlling neuronal migration might differ in lissencephalic versus gyrencephalic brains. Here we demonstrate that during late corticogenesis in ferret, reelin but not NRG1, is essential for radial-guided migration.

### Role of BLBP in neurogenesis and radial glial elongation

In addition to BLBP, intermediate filaments such as vimentin, nestin, and GFAP are radial glial markers. However their expression differs across species. GFAP is expressed in the radial processes of cells in the developing cerebral cortex of primates [70], [71], [72] while in mice, radial glia do not contain detectable levels of GFAP [73] but do express nestin [42], [74], [75]. BLBP however, is a radial glial marker expressed across species. It is found in ferrets but also in mice, rats, and humans suggesting a key role during cortical development [42], [23], [76]. An important finding of our study shows that the pattern of expression of BLBP and vimentin differs during normal cortical development in ferret. Vimentin labels radial glial processes from early development (E27) to postanal day 14 (P14) [11], [20]. BLBP is also seen at E27 but labels only cell bodies within the ventricular zone, and no processes. At E38-P0, virtually all radial glia express BLBP. Anthony et al. [77] found similar results in mice; they proposed that BLBP does not define a subtype of radial glia but rather correlates with neurogenesis. In ferrets, neurogenesis is observed until P12 [14], [12], [17]. Martinez-Cerdeno et al. [17] demonstrate that from P3 to P12, the majority of mitotically dividing cells occur in the subventricular zone in ferrets. They propose that the increase of mitosis outside the ventricular zone underlies the tangential expansion of the gyrencephalic cerebral cortex. We found that BLBP is no longer expressed in vimentin-positive radial glia at P3. This suggests that BLBP expression correlates with the bulk of ventricular mitosis responsible for the radial expansion of the cerebral cortex. A decrease of BLBP expression was observed in E24 MAM treated animals as also shown previously in reeler mice [32]. However, our model also revealed a proportion of radial glia that remain BLBP-positive and are less disrupted compared to radial glia expressing only vimentin. These results expand previous findings suggesting that BLBP is involved in radial glial elongation [42], [23]. Reelin elongates abnormal radial glia [32], [39] and upregulates BLBP [32], [78]. Similar effects have also been observed with NRG1 [23]. In our model, BLBP-positive radial glia, although substantially less disrupted, were not significantly altered when slices were exposed to reelin or NRG1. In many instances, we were successful in repairing vimentin-positive radial glia so that their morphology was comparable to the slightly disrupted BLBP radial glia; this correlated with an increase of BLBP expression (Figure 8). Our results therefore confirm that the radialization induced by reelin or NRG1 is correlated with BLBP expression but also suggest that maintaining BLBP expression might protect radial glial cells from prenatal cytotoxic injury.

### What distinguishes radial glia in MAM treated ferrets?

In normal newborn ferret, radial glial cells express vimentin as well as BLBP. In MAM treated animals at a similar age, these markers are not expressed homogeneously and about half of radial glia express vimentin but do not express detectable levels of BLBP. BLBP expressing radial glia are distinguished by being less affected by MAM treatment and less affected by attempts at repair using radializing factors such as reelin or neuregulin. During normal corticogenesis in ferrets, BLBP expression is developmentally regulated and coincides with the bulk of neurogenesis (up to P0-P1); it is not present in radial glia after about P3, while vimentin expression is maintained until P14. This suggests that BLBP is reduced as radial glia differentiate into astrocytes. MAM exposure at embryonic day 24 induces a premature differentiation of radial glia [20], [21]. Therefore, after MAM treatment, radial glial cells may exist at different developmental stages and express different levels of BLBP. In our ferret model of cortical dysplasia, early signs of radial glial differentiation are loss of pial attachment and parallel alignment as occurs in the vimentin-positive cells. This process also results in a decrease of BLBP that can be reversed by exogenous application of reelin or neuregulin1. Our data and others obtained in rodents [23], [32] indicate that reelin, neuregulin1 and BLBP are fundamental during corticogenesis in lissencephalic as well as in gyrencephalic brains. BLBP may define different stages of maturation during generation of neocortex in the ferret.

## Supporting Information

Figure S1 Schematic view of reelin and neuregulin1 signaling pathways. The pathway inhibitors and the blocking antibodies used in our study are boxed in red.
(2.25 MB TIF)
