# Supplementary figures and images for: Populations of Radial Glial Cells Respond Differently to Reelin and Neuregulin1 in a Ferret Model of Cortical Dysplasia
Source: PLoS One. 2010 Oct 28;5(10):e13709. doi: 10.1371/journal.pone.0013709 (PMC2965671; doi:10.1371/journal.pone.0013709)

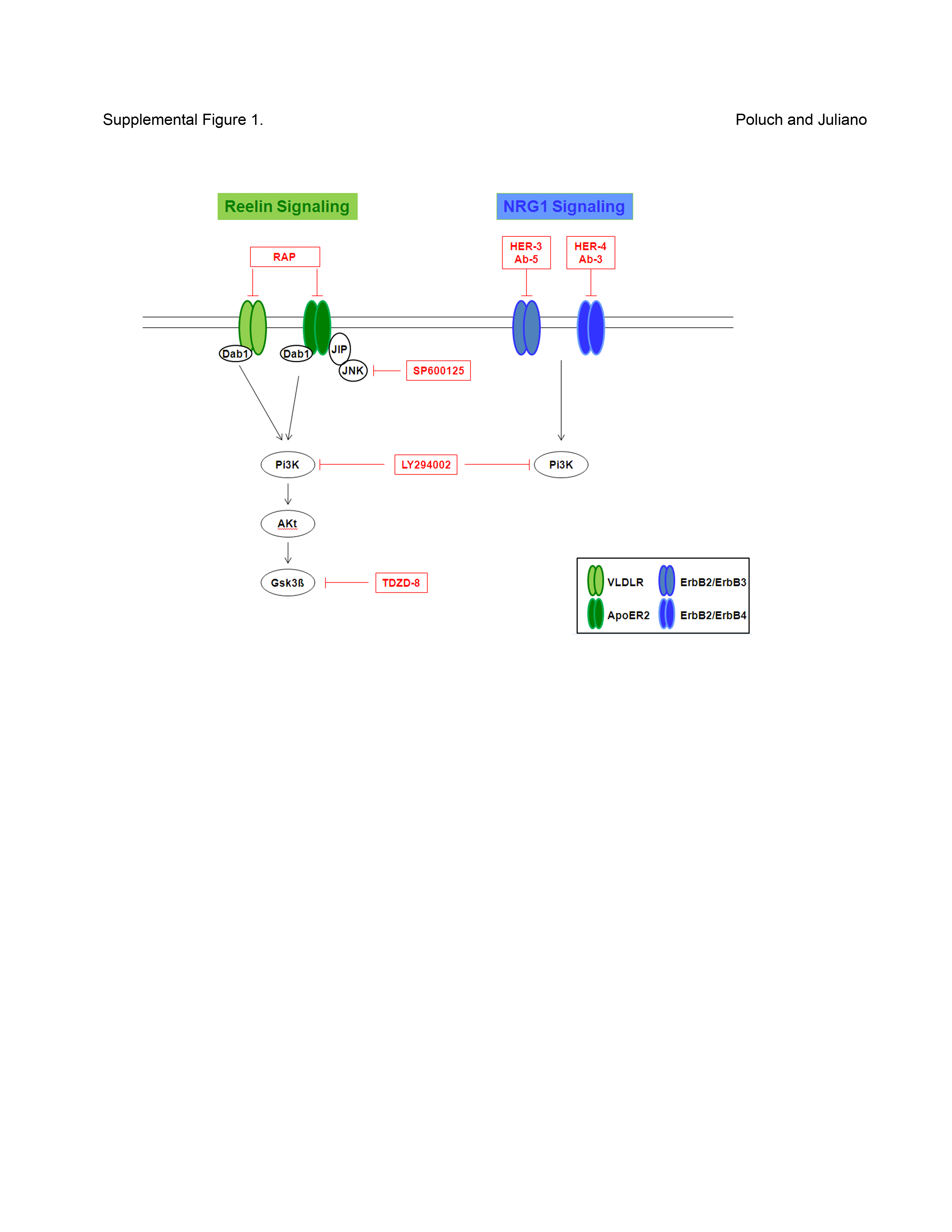

Supplement: Figure S1 — Schematic view of reelin and neuregulin1 signaling pathways. The pathway inhibitors and the blocking antibodies used in our study are boxed in red. (2.25 MB TIF) [file pone.0013709.s001.tif]
